# Processing of frequency and location in human subcortical auditory structures

**DOI:** 10.1038/srep17048

**Published:** 2015-11-24

**Authors:** Michelle Moerel, Federico De Martino, Kâmil Uğurbil, Essa Yacoub, Elia Formisano

**Affiliations:** 1 Center for Magnetic Resonance Research, Department of Radiology, University of Minnesota, Minneapolis, USA; 2 Department of Cognitive Neuroscience, Faculty of Psychology and Neuroscience, Maastricht University, Maastricht, the Netherlands; 3 Maastricht Brain Imaging Center (MBIC), Maastricht, the Netherlands

## Abstract

To date it remains largely unknown how fundamental aspects of natural sounds, such as their spectral content and location in space, are processed in human subcortical structures. Here we exploited the high sensitivity and specificity of high field fMRI (7 Tesla) to examine the human inferior colliculus (IC) and medial geniculate body (MGB). Subcortical responses to natural sounds were well explained by an encoding model of sound processing that represented frequency and location jointly. Frequency tuning was organized in one tonotopic gradient in the IC, whereas two tonotopic maps characterized the MGB reflecting two MGB subdivisions. In contrast, no topographic pattern of preferred location was detected, beyond an overall preference for peripheral (as opposed to central) and contralateral locations. Our findings suggest the functional organization of frequency and location processing in human subcortical auditory structures, and pave the way for studying the subcortical to cortical interaction required to create coherent auditory percepts.

The inferior colliculus (IC) and medial geniculate body (MGB) of the thalamus are subcortical structures that play a pivotal role in a vast number of auditory tasks. The IC is a major integration center for several brainstem pathways, and processes information that is fundamental for successful sound localization^1^. The MGB is the thalamic relay between the IC and the auditory cortex, and actively regulates the flow of information that serves as input to the auditory cortex^2^,^3^,^4^. While the IC and MGB have been examined invasively in a number of species, these nuclei, and especially subregions within these subcortical structures, have remained largely inaccessible to non-invasive investigations in the human due to their small size^5^ (IC: ~7 × 7 × 7 mm; MGB: ~4 × 5 × 4.5 mm) compared to conventional fMRI voxel volumes. Following rapid advances in the achievable spatial resolution of functional imaging, it has become feasible to obtain high spatial resolution functional images of the IC^6^,^7^ and e.g. of the lateral geniculate nucleus of the thalamus (LGN^8^,^9^). Here we take advantage of the sensitivity and specificity of ultra-high field MRI at 7T to investigate the processing in the human IC and MGB.

We explore the subcortical processing of two fundamental aspects of natural sounds. First, we investigate the processing of frequency by neuronal populations in the IC and MGB. Throughout the auditory pathway, auditory neurons can be described by their characteristic frequency (CF), which is the frequency to which a neuron responds best. At each stage of the auditory pathway neurons are topographically arranged according to their CF, resulting in one or multiple tonotopic maps. In the central nucleus of the IC, one low-to-high tonotopic gradient orthogonal to the fibrodendritic laminae has reliably been observed using invasive recordings^10^,^11^,^12^,^13^. Recent fMRI studies confirmed that this organization is preserved in the human^6^,^7^. The MGB can be divided in a ventral, dorsal, and medial part, whose tonotopic organizations have been investigated in a number of small mammals. The dorsal MGB (MGD) is not tonotopically organized, but tonotopic gradients have been observed in both the ventral MGB (MGV^14^,^15^,^16^,^17^) and medial MGB (MGM^16^,^18^,^19^). Furthermore, the posterior thalamic nucleus (Pol), which is an auditory thalamic region situated at the dorsomedial adjacency of the MGB, has been reported to contain a tonotopic gradient^20^. The presence and number of observed tonotopic gradients in the MGB differs across studies, and may be species-dependent. Exploring the frequency preference in the human MGB is important to characterize its tonotopic organization, a prerequisite for investigating thalamo-cortical interactions in human auditory processing.

Second, we explore the subcortical processing of sound location. Horizontal sound localization (i.e., localization in the azimuthal plane) relies on two binaural cues: the difference in level between the sound arriving at the two ears (i.e., interaural level difference [ILD]), and differences in arrival time (i.e., interaural time difference [ITD]^21^). In mammals, the earliest analysis of ILD and ITD cues takes place in the lateral and medial superior olive, respectively^22^. Yet, how and where in the auditory system information from spatial cues may be integrated to form a representation of horizontal space remains unclear. The IC may be particularly suited to integrate spatial cues, since it is the common target of a large number of auditory nuclei that contain diverse binaural information. An integration of spatial cues may emerge as neuronal population tuning to the full range of azimuths, possibly organized topographically. While such a map of space has been demonstrated in the IC of the barn owl^23^, evidence of a comparable map in mammals is currently lacking. Furthermore, it is interesting to investigate how subcortical processing of information on spatial location relates to the processing of the sounds’ frequency content.

Here we investigated sound frequency and location processing in subcortical auditory structures, using natural sound stimuli presented at different azimuth positions and an fMRI encoding approach^24^,^25^. Our results show that natural sound processing in IC and MGB neuronal populations were well described by a computational model that jointly encoded frequency and location preference. That is, subcortical auditory neuronal populations could be characterized by their frequency-specific coding of sound location. Based on the estimated weights of the trained joint frequency-location model, topographic maps of preferred frequency and location preference were generated. Frequency tuning was organized in one dorsolateral to ventromedial low-to-high tonotopic gradient in the human IC. A mirror-symmetric low-high-low (from dorsomedial to ventrolateral locations) tonotopic pattern characterized the auditory thalamus, reflecting two MGB subdivisions. Both the IC and MGB displayed a preference for contralateral (over ipsilateral), and for peripheral (over central) sound locations. No topographic map of preferred azimuth could be identified.

## Results

### Subcortical responses to natural sounds

We collected fMRI responses at 7T while subjects (*N* = 6) listened to 84 natural sounds (including human speech, animal cries, and tool sounds). Spatial cues were included in these sounds by recording them for each volunteer separately in a virtual reality lab (see Supplementary Fig. 1, and Methods). Each sound was played at one of 7 frontal azimuthal locations, ranging from the left (−90°) to the right (90°) of the subject in 30° steps (0° elevation). An analysis of the recorded sounds revealed the presence of ITD cues only in lower frequency ranges (between 0.2 and 1.5 kHz), while ILD cues were informative for all frequencies above 0.3 kHz (Supplementary Fig. 1c). ILD information in low frequency ranges was likely induced by our experimental setup^26^ (i.e., recordings were not free-field, but made in a normal room with reverberations).

We observed responses to the sounds bilaterally in the IC and MGB of each individual and at the group level (see Supplementary Fig. 2). In some subjects the response to the sounds extended to voxels inside the superior colliculus (SC; see for example coronal IC slice of S6 in Supplementary Fig. 2a). However, as activation of the SC was much smaller than that of the IC and only present in a subset of volunteers, it was excluded from further analysis.

### Model comparison

**Figure 1:**
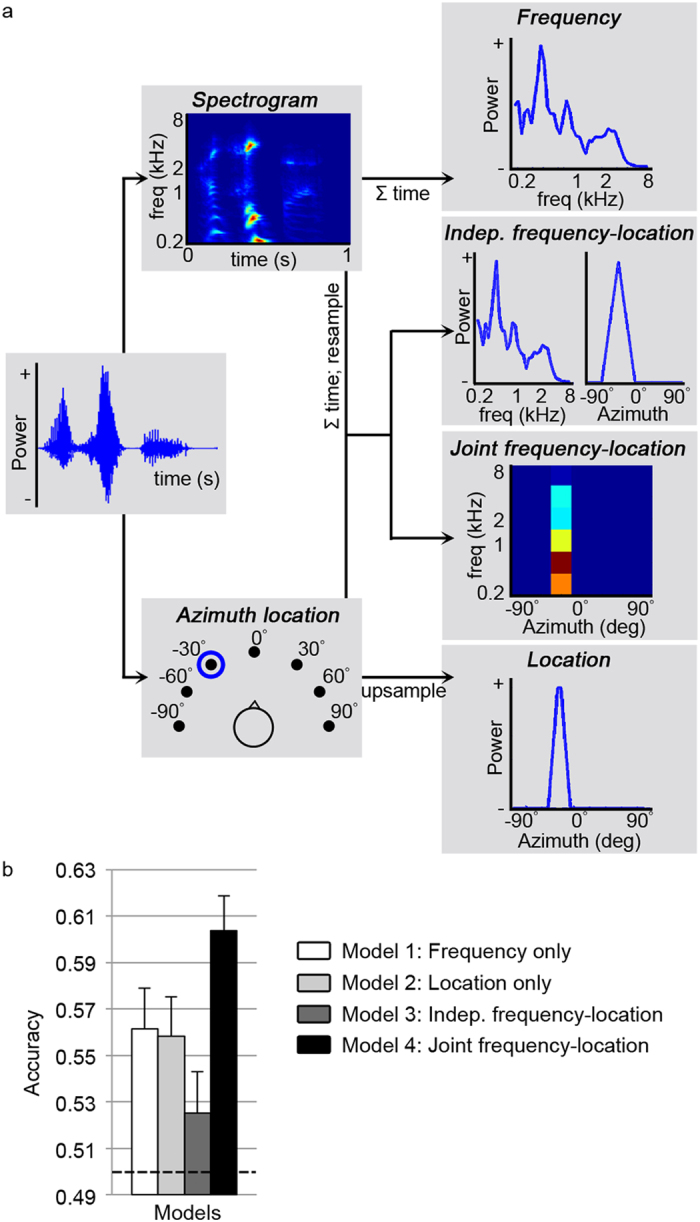
Overview and performance of encoding models. (**a**) The sounds were represented by four computational models. Top: The sound representation in the *frequency only* model space was obtained by averaging the sound’s spectrogram over time. Second row: For the *independent frequency-location* model, the representation of the sound in the frequency space was concatenated with a vector representing the sound’s location. Third row: The *joint frequency-location* model was obtained by setting the bins representing the sound’s location to the values reflecting the sound’s frequency content. Bottom: The stimulus representation in the *location only* model space was obtained by upsampling a vector representing the sound’s location. (**b**) Bars indicate the prediction accuracy for the four models across subjects (mean + s.e.m., *N* = 6). The *frequency only*, *location only* and the *joint frequency-location* model performed significantly above chance. However, the joint frequency-location model was significantly better at representing the fMRI data than the single feature models (see Supplementary Fig. 3 for individual subject results). Chance level is 0.5 and is indicated by the dashed black line.

We analyzed the observed responses to natural sounds with an encoding approach, and compared four computational models of subcortical processing. Each computational model represents a hypothesis about how frequency and sound location are informative in explaining the fMRI responses in subcortical voxels. Specifically, we compared single feature models’ encoding for frequency^27^ or sound location separately, to combined models in which the two features were independently or jointly coded (see Fig. 1a and Methods). While performance of the single feature models is informative on whether a feature is useful in explaining subcortical responses to natural sounds, comparing different combined-feature models to each other provides information on the relation between the represented features (frequency and azimuthal location).

Model training and testing was done per subject, combined for the IC and MGB, as follows. All models were trained on a subset of the data (56 ‘training’ sounds and corresponding fMRI responses). The model features of each training sound (e.g., the training sounds’ frequency content for the frequency only model) were used to explain fMRI response (i.e., beta values) to all training sounds using a regularized regression analysis. This ‘training’ step resulted in a weight vector (per voxel) representing the preference of each voxel for each model feature. This trained model was evaluated using a sound identification analysis^24^,^28^. That is, the weight vector (per voxel) was used to predict responses to 28 testing sounds (not used during the training), thereby testing the generalization of the learned model to unseen data. Comparing prediction accuracies across models revealed which model (i.e., computational hypothesis) best represented the fMRI data. Importantly, based on the weights of the trained best model, topographic maps of preferred frequency and location preference could be generated (see below).

The *independent frequency-location* model did not predict responses to test sounds significantly above chance. Instead, the *frequency only*, the *location only* and the *joint frequency-location* model performed significantly above chance (chance = 0.5) at the group level (*N* = 6; frequency only: mean [s.e.m.] = 0.56 [0.02], *p* = 0.010; location only: mean [s.e.m.] = 0.56 [0.02], *p* = 0.011; joint frequency-location model: mean [s.e.m.] = 0.60 [0.02], *p* = 0.001, one-tailed one-sample t-tests on Fisher transformed prediction accuracy values, corrected for multiple comparisons; see Fig. 1b). Above chance performance of the *frequency only* and *location only* model indicates that both sound features were represented subcortically. However, the *frequency only* and *location only* models performed significantly worse than the *joint frequency-location* model (*p* = 0.041 and p = 0.018, respectively, one-tailed paired t-test on Fisher transformed prediction accuracy values; see Fig. 1b). Furthermore, the *joint frequency-location* model predicted responses to novel sounds significantly above chance in 5 out of 6 subjects (alpha level = 0.05; based on permutation testing; see Supplementary Fig. 3). The superior performance of the joint frequency-location model indicates that a model including both frequency and location information was a better representation of the collected fMRI data than a model based on either of these features alone. However, location information only contributed to the representation of fMRI data if it was included in a frequency-specific manner. That is, the model had to consist of *combined* frequency-location filters (as opposed to independent frequency and location filters).

### Frequency tuning and tonotopic gradients

**Figure 2:**
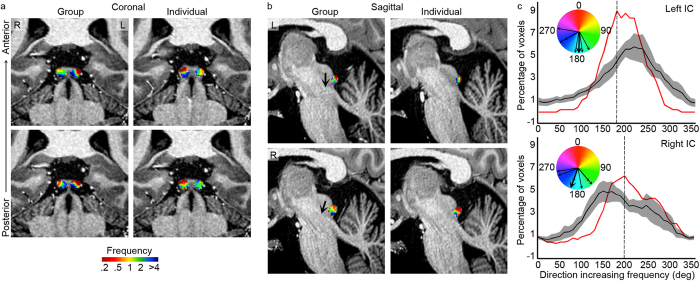
Group and individual tonotopy in the IC. (**a,b**) Maps of frequency preference (tonotopic maps) in the group (first column) and in an individual (S1; second column), within regions responding significantly to the natural sounds. Coronal (**a**) and sagittal (**b**) views of the maps are superimposed on high-resolution anatomical images of the individual subject. Coronal views of two locations on the anterior-posterior axis are shown from top to bottom, and for the sagittal views a cut through left (top) and right (bottom) IC are displayed. (**c**) Distribution of direction of increasing frequency, calculated on individual voxels in sagittal slices (4 slices for the left and right IC each). Red and black lines show the direction of increasing frequency for the group tonotopy map and the average of distributions resulting from individual maps, respectively (±s.e.m. in grey). Black arrows in the coloured circles represent the peak of red curve (thick arrow, representing the main direction of increasing frequency in the group tonotopy map), and peaks of the individual distributions (thin arrows, representing the main direction of increasing frequency in the individual tonotopy maps; subjects with the same peak value are displayed as additional arrowheads).

Next, we generated maps of preferred frequency and location based on the weight vectors estimated using the *joint frequency-location* model. That is, each voxel was colour-coded according to the frequency and location with the highest weight in the trained model. Given the small size and variability in the anatomically-defined MGB across subjects, we aligned the IC and MGB anatomically before analyzing and interpreting the topographic maps. In accordance with previous observations^6^, we observed a single tonotopic gradient in the IC (see Fig. 2a,b). The low-to-high gradient ran in dorso-lateral to ventro-medial direction (i.e. from preference for low frequencies in superior-posterolateral locations, to preference for high frequencies in inferior-anteromedial locations). We quantified the group and individual tonotopic gradient by computing the direction of increasing frequency in a sagittal slice (4 slices for the left and right IC each). The resulting distributions were unimodal, confirming the visual observation of one tonotopic map (Fig. 2c). The main directions of increasing frequency were 180° and 200° in the mean maps (peak of red lines in Fig. 2c), and 218.3° [±20.3°] and 193.3° [±23.6°] as averaged [±s.e.m.] across individual subject distributions for the left and right IC, respectively. These directions of increasing frequency are consistent with those observed in our previous study^6^.

**Figure 3:**
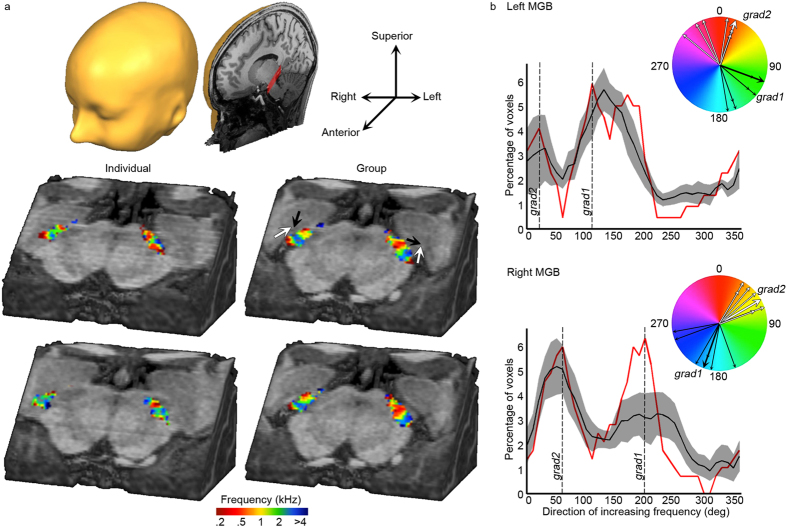
Group and individual tonotopy in the MGB. (**a**) Maps of frequency preference (tonotopic maps) in two individual subjects (first column; see Supplementary Fig. 4 for all individual maps) and in the group (second column). A cube of brainstem based on a high-resolution anatomical image of an individual subject is shown from the front-left, and a cut through it is made at a 45° angle such that the MGB is visible (indicated as the red shaded area on the cut in the left upper corner inset). The maps are superimposed on this image. For the right column, from top to bottom superior to inferior cuts through the MGB are displayed. (**b**) Distribution of direction of increasing frequency, calculated on individual voxels in slices oriented as in (**a**). Red and black lines show the direction of increasing frequency for the group tonotopy map and the average of distributions resulting from individual maps, respectively (±s.e.m. in grey). Black and white arrows in the coloured circles represent the first and second peak of red curve (thick arrow, representing the two main directions of increasing frequency in the group tonotopy map), and two peaks of each individual’s distribution (thin arrows, representing the main directions of increasing frequency in the individual tonotopy maps; subjects with the same peak value are displayed as additional arrowheads).

Figure 3a displays group and a subset of individual maps of frequency preference in the MGB (maps of all individual subjects are shown in Supplementary Fig. 4). In the group MGB tonotopy maps, two low frequency regions could be identified at the superior-posterior-medial end and inferior-anterior-lateral end of the map respectively (red colours in right column of Fig. 3a). In between these low frequency clusters, voxels were tuned to higher frequencies (blue colours in right column of Fig. 3a), resulting in a low-high-low gradient. We quantified the group and individual tonotopic gradients by computing the direction of increasing frequency. This computation was performed on the slices as displayed in Fig. 3a (i.e., on a ventro-dorsal cut through the brainstem, at a −45° angle with respect to the anterior-posterior commissure axis; 3 slices for the left and right MGB each). The resulting distributions were bimodal, confirming the visual observation of two tonotopic maps (Fig. 3b). The dorsal low-to-high gradient (indicated with black arrows in Fig. 3) had a main direction of increasing frequency of 110° and 200° in the mean tonotopy map (peak of red lines in Fig. 3b), and 143.3° [±10.5°] and 211.7° [±6.8°] as averaged [±s.e.m.] across individual subject distributions for the left and right MGB, respectively. The ventral low-to-high gradient (indicated with white arrows in Fig. 3) had a main direction of increasing frequency of 20° and 60° in the mean tonotopy map (peak of red lines in Fig. 3b), and 351.7° [±13.1°] and 48.3° [±8.2°] as averaged [±s.e.m.] across individual subject distributions for the left and right MGB, respectively. Table 1 shows the Talairach COM coordinates of the low and high frequency clusters, listing a superior-posteromedial ([x y z] = [12.5 −25.5 −2.7]) and inferior-anterolateral ([x y z] = [15.5 −23.2 −4.1]) low frequency cluster with a high frequency cluster in intermediate locations.

### Distribution and maps of preferred azimuth

**Figure 4:**
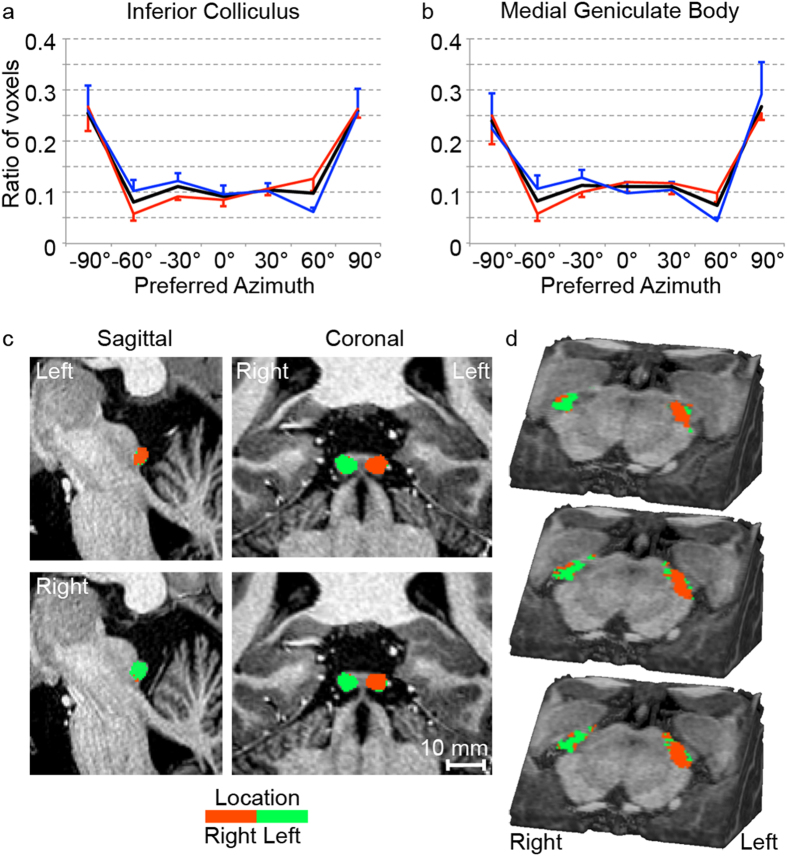
Spatial location preference in the IC and MGB. (**a,b**) The ratio of voxels in IC (left) and MGB (right) tuned to each of the locations (mean ± s.e.m.). Black lines show the average across all voxels, while red and blue lines display location tuning separately for voxels with a low CF (<1.5 kHz) and high CF (>1.5 kHz), respectively. In both subcortical structures, a significantly larger part of the voxels preferred the extreme locations (−90° to 90°), reflecting sounds presented on the complete left and right respectively, compared to the other locations. No significant differences were observed between the part of voxels tuned to each of these other locations (−60° to 60°). (**c, d**) Maps of azimuth preference (tuning to spatial location) at the group level in the IC and MGB respectively. (**d**) A cube of brainstem based on a high-resolution anatomical image of an individual subject is shown from the front-left, and a cut through it is made at a 45° angle such that the MGB is visible. The maps are superimposed on this image. From top to bottom, superior to inferior cuts through the MGB are displayed.

Subsequently, we explored the distribution of preferred azimuths, combined across left and right subcortical structures. As location tuning may be different for voxels preferring low and high frequencies (voxels with CF < 1.5 kHz were informed by ITD and ILD; voxels with CF > 1.5 kHz were informed exclusively by ILD), we also investigated the distribution of preferred azimuth separately for voxels with a low (<1.5 kHz) and high (>1.5 kHz) CF. While approximately 10% of voxels preferred each of the azimuths ranging from −60° to 60°, a significantly higher number of voxels was tuned to the extreme left (−90°) and extreme right (90°; see black lines in Fig. 4a,b; alpha level = 0.05 in both IC and MGB; *N* = 6; one-tailed paired t-test corrected for multiple comparisons). No changes with respect to this overall pattern were observed when considering the distribution of azimuth tuning for low and high frequency preferring voxels separately (red and blue lines in Fig. 4a,b, respectively).

We examined the spatial arrangement of location tuning in the IC and MGB. Both structures displayed a contralateral bias (Fig. 4c,d), such that the left IC/MGB preferred azimuthal locations on the right (shown in orange) and the right IC/MGB preferred azimuthal locations on the left (shown in green). Within each individual IC and MGB, substantial regions of ipsilateral tuning could be observed as well (see Supplementary Fig. 4). While tuning to ipsilateral locations tended to occur at the periphery of both IC and MGB, the spatial arrangement of contra- vs. ipsilateral tuning was not consistent across subjects. Finally, we explored maps of tuning to each of the contralateral and ipsilateral spatial locations to examine the presence of a topographic map of space (Supplementary Fig. 5). No topographic map of preferred azimuth could be detected. Note that this by itself does not preclude the existence of a map of space, as this finding could be due to possible limitations in the stimuli, design, and spatial resolution of our study.

## Discussion

We used high-resolution fMRI to investigate the representation of sound frequency and sound location in the human IC and MGB. By showing above chance performance of a model that includes only frequency or only azimuthal location information, we have demonstrated that both frequency and sound location information are encoded in the IC and MGB. The superior performance of the *joint frequency-location* model indicates that location information only contributes to the representation of fMRI data if it is included in a frequency-specific manner (and vice versa). That is, the model must consist of *combined* frequency-location filters (as opposed to independent frequency and location filters). This result implies that an examination of subcortical tuning to azimuthal location should sample a full range of frequencies in order to draw a valid conclusion about tuning to location. Furthermore, it suggests that at the level of subcortical neuronal populations, azimuth information is not yet integrated along the whole range of frequencies. It would be interesting to extend these explorations to the auditory cortex and compare model performance throughout core, belt, and parabelt cortical processing stages. As previous studies reported the processing of spectrotemporal modulations in the IC^29^,^30^, extending the computational models to represent these processing aspects is a next step in the exploration of subcortical processing. Adding relevant features to the computational models, in addition to further increasing the spatial resolution (for example by collecting data at 9.4T), may improve model performance in future studies.

Before interpreting the topographic maps, a note of caution must be given. Functional MRI is an indirect measure of neuronal activity, originating from changes in blood oxygenation, flow, and volume. It is spatially limited, and may be too coarse to accurately discriminate topographic patterns even at the high resolution at which we acquired the data. The measured fMRI signal depends on the vascular morphology, which is not well defined for the IC and MGB. Theoretically, draining veins could shift the location of observed preferences, tilting observed mappings or biasing resulting maps to specific frequency or location preferences^31^. In the current study, these confounds are partially mitigated by the relatively higher sensitivity of gradient echo BOLD to the micro-vasculature at 7T^32^ compared to lower fields. Future studies could reduce this concern even further by using high field spin echo BOLD, as it exhibits reduced sensitivity to larger vessels^31^. However, whether there is enough sensitivity in the midbrain to generate these maps using spin echo BOLD is unclear.

**Figure 5:**
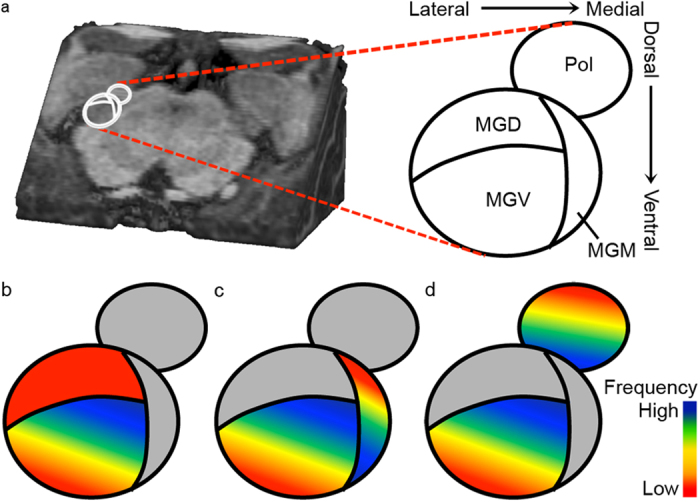
Interpretation of the MGB tonotopic gradient. The cartoon reflects the right auditory thalamic regions as a 45° cut through the brainstem, comparable to the views in Figs 3a and 4d. (**a**) Dorsal and ventral directions reflect posterior-superior and inferior-anterior locations respectively. Abbreviations indicate the relative locations of the ventral (MGV), dorsal (MGD), and medial (MGM) division of the MGB, and the posterior thalamic nucleus (Pol). (**b–d**) We interpret the ventral high-to-low part of the observed tonotopic gradient as reflecting the dorsomedial to ventrolateral tonotopic map in MGV. The dorsal low frequency region could alternatively reflect (**b**) low frequency tuning in MGD, (**c**) a low-to-high dorsomedial to ventrolateral tonotopic map in MGM, or (**d**) a low-to-high dorsomedial to ventrolateral tonotopic map in Pol.

Our results show one robust tonotopic gradient in the IC, and a low-high-low map for the MGB. Support for the presence of a tonotopic gradient in the central nucleus of the IC comes from a variety of non-human species using invasive electrophysiological recordings^10^,^11^,^13^,^33^. Noninvasive fMRI studies have confirmed the presence of this tonotopic gradient, both in the monkey^30^ and in the human^6^,^7^. Our observation of a dorsolateral to ventromedial low-to-high tonotopic gradient in the IC endorses these previous findings. While MGB frequency preference has been studied invasively in a number of non-human species, to the best of our knowledge this is its first investigation in the human. The ventral low-to-high part of the tonotopic gradient most likely reflects the ventral division of the MGB (MGV; ‘grad2’ and white arrows in Fig. 3; see Fig. 5). Previous studies in a variety of non-human species conclusively support the presence of a tonotopic gradient in ventral MGB (cat^14^,^17^; rabbit^15^; mouse^16^). Throughout species, the tonotopic axis runs orthogonal to the laminated fibrodendritic structure present in MGV^14^,^15^,^34^. While no functional tonotopic explorations have been reported in the human, a cytoarchitectonic study described the occurrence of “large neurons [that] formed clusters surrounded by a particular pattern of neuropil which, together, constituted fibro-dendritic laminae whose long axis was oriented mediolaterally in parallel sheets or rows” in human MGV^5^. Thus, the main tonotopic gradient in the human MGV may be expected to run orthogonal to this laminar pattern: in ventral-to-dorsal direction. Our maps partially confirm this prediction, showing a ventral-to-dorsal but also a lateral-to-medial component to the low-to-high tonotopic gradient (see Figs 3 and 5).

The dorsomedial region of low frequency preference in our tonotopic maps presumably lies outside the ventral division of MGB, and below we propose three alternative interpretations for this region (see Fig. 5b–d; ‘grad1’ and black arrows in Fig. 3). First, the region may reflect a low frequency preference in the dorsal division of the MGB (MGD; see Fig. 5b). However, neither the tuning to intermediate frequencies in dorsal MGB locations, nor the medial location of the dorsal low frequency region compared to the ventral one, can be explained based on this hypothesis. A second option is that this region reflects a tonotopic gradient within the MGM (see Fig. 5c). Previous studies lend support to the existence of a (weak) tonotopic map in the MGM (cat^18^,^19^; mouse^16^). While this second hypothesis is not in line with the observed discrepancy between tonotopic maps and the siT_1_-defined MGB (i.e. in this hypothesis the functional responses occupy the full medio-lateral extend of siT_1_-defined MGB, while we observed a lateral position of the functional responses with respect to the siT_1_-based definition of MGB; see Methods and Supplementary Fig. 6), it is consistent with the tonotopic maps. Third, the dorsomedial region of low frequency preference in our tonotopic maps may reflect an auditory region beyond the MGB; the lateral part of the posterior thalamic nucleus (Pol; see Fig. 5d for a schematic representation of this interpretation). Pol is traditionally considered as belonging to the secondary (i.e. non-lemniscal) auditory pathway (but see^35^), yet is tonotopically organized^18^. Its high frequency region (but not its intermediate or low frequency region) was reported to be “contiguous to the high frequency region of MGV”^20^ consistent with the tonotopic pattern present in our maps. As Pol is located superior to the MGB and our entire functional map is contained within the siT_1_-defined MGB, a consequence of this third hypothesis is that we must interpret Pol as situated inside the siT_1_-defined MGB. Further explorations combining a multisensory functional localizer with an array of anatomical measures *within the same individuals* are needed to determine the likelihood of an inclusion of Pol within the siT_1_-defined MGB, and an inclusion of multisensory region MGM^36^,^37^ in the tonotopic maps. In conclusion, while an interpretation of the ventral tonotopic part as MGV is well supported, further anatomical and functional explorations are needed to decide between the alternative interpretations of the dorsal part of the tonotopic MGB gradient.

The observed bias to contralateral sound locations in IC and MGB is consistent with results from previous human fMRI studies^38^,^39^,^40^ and electrophysiological recordings in mammals^41^,^42^,^43^,^44^,^45^. Beyond contralateral tuning, we did not observe a map of space. Even at the high resolution of our measurements, it may be that the map of space is organized at a resolution finer than accessible by our protocol. Future work, following advances in attainable spatial resolution with fMRI, may reveal this.

To date, it remains unclear how ILD and ITD cues are represented in human subcortical structures, and how they are combined to form a representation of horizontal space. While our experimental setup had several advantages (i.e., natural sounds elicit robust subcortical fMRI responses; the use of individually recorded sounds generated a strong percept of auditory space in the scanner), it did not allow a separate analysis of the representation of ILD vs. ITD cues. Specifically, due to the reverberations in the virtual reality lab^26^ ILD cues were present in low frequency bands, removing the exclusive relation between low frequencies and ITD cues.

Invasive recordings in small mammals showed that the IC contains neurons that are tuned to ILD, a portion of which is invariant to the average binaural level (ABL)^43^,^46^. For both low and high frequency preferring voxels, we observed neuronal populations tuned to each azimuthal location, and a relative abundance of tuning to the periphery (−90° and 90°; Fig. 4a,b). High frequencies (>1.5 kHz) were exclusively informed by ILD cues, and our results therefore support that approximately 60% of the high frequency preferring human IC and MGB are tuned to ILD’s corresponding to the periphery (−90° and 90°), and 40% to frontal locations (ranging between −60° and 60°). However, these estimates must be interpreted with great caution. First, all sounds were presented at an equal and relatively high intensity and it cannot be excluded that neuronal populations show a different pattern of location tuning at lower sound intensity. Second, even at the high spatial resolution achievable with 7T, each voxel measured the pooled activity of a large number of neurons. A voxel that sampled neurons tuned to each peripheral location may display tuning to their average (a frontal location). Alternatively, fine-grained location tuning present at the single neuron level, if surrounded by differently tuned neurons, may average out at the voxel’s population level.

Two opposing theories have been hypothesized to describe the coding of ITD. The traditional view, originally proposed by Jeffress^47^, holds that ITD is coded according to a ‘peak’ strategy (or ‘place code’) in which neuronal populations each respond most vigorously to a specific location. At the population level, the preferred locations cover the range of possible sound locations in the environment, and readout occurs as the weighted mean activity of neuronal populations. Alternatively, the ‘Opponent Channel’ model^48^,^49^ proposes a population coding strategy (i.e. ‘rate code’), in which a sound’s location is estimated based on the relative activity within two populations of neurons that are broadly tuned to either the left or right auditory space. Within this strategy, the slope (as opposed to the peak) of the neuron’s tuning curve provides the most spatial information. The majority of neuronal populations with open-ended response patterns as observed for voxels with low frequency preference in the current study (Fig. 4a,b), is both predicted and required by the ‘Opponent Channel’ model. Our data therefore favor this model over the Jeffress model. However, the limitations mentioned above (i.e., sound level, spatial resolution of fMRI) must be kept in mind when interpreting this result. Moreover, as ILD cues were present in all frequency bands, the relative abundance of peripheral tuning in low frequency bands may be driven by ILD tuning^50^. Future work, using artificial sounds that are varied exclusively in ITD or ILD (i.e., not individually recorded, but carefully manipulated), together with the fMRI methods described in the current study, are needed to investigate the processing of each of these location cues.

## Methods

### Ethics statement

The experimental procedures were approved by the ethics committee of the Faculty for Psychology and Neuroscience at Maastricht University, and were performed in accordance with the approved guidelines and the Declaration of Helsinki. Informed consent was obtained from each participant before conducting the experiments.

### Subjects

Six subjects participated in this study (mean age [SD] = 25.0 [1.7]; one male and five females). The subjects had no history of hearing disorder or neurological disease.

### Stimuli

The stimuli consisted of recordings of 84 natural sounds (including human speech, animal cries, and tool sounds). Sounds were sampled at 16 kHz and their duration was 1000 ms. Sound onset and offset were ramped with a 10 ms linear slope, and their energy (RMS) levels were equalized.

Spatial cues were included in the sounds by recording them for each volunteer separately in a virtual reality lab (Ambisonic 3D Auralizer system, Worldviz). The lab had an internal volume of 95 m^3^. The walls and ceiling consisted of gypsum board and the floor consisted of wood covered with a thin carpet. The lab was equipped with 22 speakers arranged in a sphere around the participant in the far field (12 speakers in the horizontal plane at the elevation of the interaural axis [vertical azimuth 0°; distance of 2.4 m from the participant] and 5 speakers above and below vertical azimuth 0° respectively). Virtual reality software (Vizard Worldviz, http://www.worldviz.com/) was used to position sounds in the acoustic 3D environment. Subjects were seated in the middle of the circle and were outfitted with two inner ear recording devices (OKM II classic microphone; http://www.soundman.de/en/products/okm-ii-studio-rock/). To minimize head movement, the subject was asked to focus on a frontal fixation cross. Each sound was played and recorded in 7 frontal azimuthal locations, ranging from the left (−90°) to the right (90°) of the subject in 30° steps (0° elevation).

This recording procedure created realistic, well localizable natural stimuli. We analyzed the ILD of each recorded sound as the difference in power between the sound’s spectrogram at the left and right ear. ITD was computed by converting the interaural phase differences (extracted from the frequency-time spectrograms at the left and right ear) to time differences. We computed ILD and ITD information per frequency bin, by subtracting the ILD (or ITD) at +90° from that at −90°, and normalizing resulting values (division by the maximum). Note that positive values indicated that the ILD or ITD cue was informative. Statistical significance of ILD and ITD information was tested with a one-tailed one-sample t-test per frequency bin (*p* < 0.05; corrected for multiple comparisons).

### Anatomical MRI data

Data were acquired on an actively shielded MAGNETOM 7T whole body system driven by a Siemens console at Scannexus (www.scannexus.nl). The magnet had a body gradient insert operating at 70 mT/m with a slew rate of 200 T/m/s. A Nova Medical head RF coil (single transmit, 32 receive channels) was used to acquire anatomical (T_1_, Proton Density [PD], and short inversion T_1_ [siT_1_] weighted) and functional (T_2_* weighted BOLD) images. T_1_ weighted (0.6 mm isotropic) images were acquired using a modified MPRAGE sequence (repetition time [TR] = 3100 ms; time to inversion [TI] = 1500 ms; echo time [TE] = 2.52 ms; flip angle = 5°; generalized autocalibrating partially parallel acquisitions [GRAPPA] = 3; field of view [FOV] = 229 × 229 mm; matrix size = 384 × 384; 256 slices; pixel bandwidth = 181 Hz/pixel). PD images were acquired with the same MPRAGE as the T_1_ weighted image but without the inversion pulse (TR = 1140 ms; TE = 2.52 ms; flip angle = 5°; GRAPPA = 3; FOV = 229 × 229 mm; matrix size = 384 × 384; 256 slices; pixel bandwidth = 181 Hz/pixel), and were used to minimize inhomogeneities in T_1_ weighted images^51^. Acquisition time for the T_1_ and PD datasets were ~9 and 4 minutes respectively.

While the posterolateral borders of the IC are clearly visible in standard anatomical images due to their distinctive shape, MGB *cannot* be identified in conventional anatomical images. Instead, MGB outlines, along with the outlines of a large number of thalamic nuclei, can be seen in a T_1_ weighted image where the inversion time is modified to null white matter and thereby enhance grey matter contrast (short inversion time T_1_^52^ [siT_1_]). We collected a siT_1_ dataset (0.6 mm isotropic) for each subject in a separate session. Parameters were set based on a previous study^52^ (TR = 4500 ms; TI = 670 ms; TE = 3.46 ms; flip angle = 4°; GRAPPA = 3; FOV = 229 × 229 mm; matrix size = 384 × 384; 256 slices; pixel bandwidth = 178 Hz/pixel; acquisition time = ~9 minutes). The shorter TR compared to previous work^52^ was chosen as a compromise between total acquisition time and SNR. In this second session, an additional T_1_ and PD dataset were acquired and used to align the siT_1_ dataset to the functional and anatomical data collected in the first session. Anatomical data were analyzed with BrainVoyager QX and were resampled (with sinc interpolation) in the normalized Talairach space^53^ at a resolution of 0.5 mm isotropic.

### Anatomical identification of the auditory thalamus

**Figure 6:**
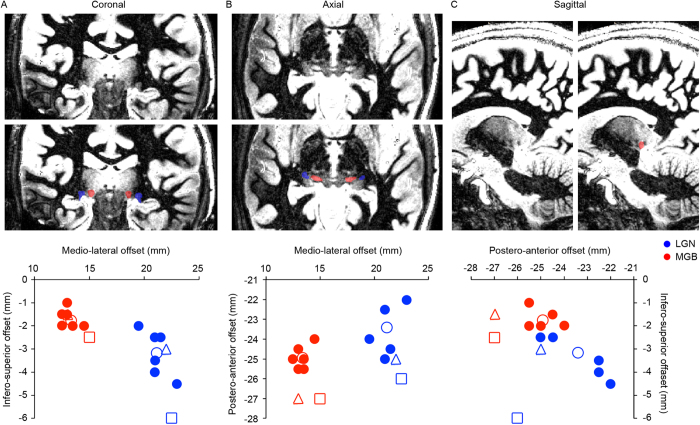
Anatomical delineation of LGN and MGB. (**a**–**c**) Coronal, axial, and sagittal view of an individual siT_1_ dataset (data from S1). The LGN was defined as the high intensity inverted teardrop shaped structure in coronal images ((**a**), in blue), and the MGB was identified as a high intensity oval shape in axial slices ((**b**), in red). (**d**) Scatterplots of the Talairach center of mass (COM) coordinates of the MGB (in red) and the LGN (blue). Filled and open circles represent individual coordinates and the mean across subjects respectively. The triangle represents the siT_1_-based coordinates as observed by a previous study^52^, and the square shows the thalamic nuclei as defined in the Morel histological atlas^56^. The origin (0, 0, 0) is at the middle of the anterior commissure.

Based on the siT_1_ dataset, we segmented both the lateral geniculate body (LGN) and MGB in native space of each individual (0.6 mm isotropic; delineation performed by MM; see Fig. 6). While the MGB segmentation was later used in the analysis to align datasets across subjects, LGN segmentation only served as a quality control of the siT_1_ parcellation. Segmentation was performed by following the descriptions of the thalamic nuclei in previous work^54^. The LGN was identified as a high intensity region with a characteristic inverted teardrop shape visible in coronal slices (see blue shapes in Fig. 6a). The MGB was identified in axial slices, where it was visible as an oval shape with the long axis in (postero-)medial to (antero-)lateral direction (see red shapes in Fig. 6b). After manual delineation, which was performed on both left and right LGN/MGB, the segmented nuclei were resampled (with sinc interpolation) at a resolution of 0.5 mm isotropic and brought to the normalized Talairach space. The x-coordinate of the left LGN/MGB was multiplied by -1 and coordinates were averaged across the left and right side of the brain.

The center of mass (COM) coordinates of these MR-defined nuclei were then compared to those based on an electronic version of the Morel histological atlas^55^,^56^, to the siT_1_-based coordinates as observed by a previous study^52^, and to the COM of individual functional responses (overall response to the sounds computed as described below; maps smoothed with a Gaussian filter [FWHM = 4 voxels], *p* < 0.05 uncorrected). Overall, the mean (across subjects) Talairach center of mass (COM) coordinates of both LGN ([x y z] = [21 -23 -3]) and the MGB ([x y z] = [13 -25 -2]) corresponded well with the previously reported coordinates^52^ and with the classic Morel histological atlas^55^ (see Fig. 6). For the MGB, a small discrepancy both to previously reported coordinates^52^ and to the Morel histological atlas of ~2 mm was present in the anterior-posterior direction. The volume of the siT_1_-defined MGB (mean [s.e.m.] = 129.4 [10.7] and 123.4 [14.3] mm^3^ for left and right MGB, respectively) was larger than the reported size of human MGB based on histology^5^,^57^ (5 mm wide × 4 mm deep × 4-5 mm long = 90 mm^3^, and 73 and 72 mm^3^ for left and right MGB respectively). No significant difference was observed between the volume of the left and right siT_1_-defined MGB.

Functional responses occupied the lateral and inferior part of the siT_1_-defined MGB. This relationship between functional and anatomical results held across variations present at the level of individual subjects (see Supplementary Fig. 6). The Talairach COM coordinates of functional responses in individual subjects, computed as the average of the locations of those voxels that responded significantly to the natural sounds and weighted by the strength of the voxels’ responses, confirmed this visual observation ([x y z] = [14-25-3]). Thus, the siT_1_-dataset may have identified a region that is larger than MGB proper. Alternatively, it is also possible that previous cytoarchitectonic explorations^5^,^57^ underestimated the MGB volume and that the natural sounds in our paradigm did not drive the complete MGB.

### Functional MRI data

T_2_* weighted functional data were acquired using a clustered Echo Planar Imaging (EPI) technique. The experiments were designed according to a fast event-related scheme. The acquisition parameters were: TR = 2800 ms; time of acquisition [TA] = 1600 ms; silent gap = 1200 ms; TE = 19 ms; echo spacing = 0.8 ms; GRAPPA = 2; partial Fourier 6/8; FOV = 132 × 132 mm; matrix size = 120 × 120; number of slices = 28; voxel size = 1.1 × 1.1 × 1.1 mm^3^. The acquisition parameters were optimized for imaging the small subcortical structures by decreasing the field of view and acquiring data in the anterior-posterior phase encode direction. This allowed for increased efficiency, higher SNR, and reduced image distortions. It also created wrapping artifacts (i.e. the front of the brain was aliased on the back and vice versa), but this did not affect the middle of the brain. Contaminated data was removed before starting the data analysis. In addition to the subcortical structures, our slices covered the inferior part of the auditory cortex. However, we focused exclusively on the IC and MGB, as the analysis of cortical responses was not part of this study.

The sounds were divided into training and testing sets (56 and 28 sounds respectively). Training sounds were presented once per training run, with a total of 6 training runs. Testing sounds were presented 3 times per run, for two runs in total. Each sound was presented in one of 7 azimuthal locations (ranging from the left [−90°] to the right [90°] of the subject in 30° steps). Semantic sound category was balanced across azimuthal locations, such that there were three voice, speech, animal, and tool sounds per location (two sounds per category for training, one for testing). Sounds were the same across subjects, yet the location at which each sound was presented was randomized across subjects. This was done to ensure that across subjects the voxels’ estimated preference to azimuthal location, as calculated later in the analysis, would not be confounded with voxels’ preference for any other sound feature varying consistently across locations. Within each run, sounds were randomly spaced at a jittered interstimulus interval of 2, 3, or 4 TRs and presented in the middle of the silent gap between acquisitions (leaving 100 ms of silence before and after the sound). Zero trials (trials where no sound was presented [5% of the trials]), and target trials (trials in which a sound was presented in the same location [5% of the trials]) were included. Subjects were instructed to perform a one-back task, and were required to respond with a button press when two consecutive sounds were presented in the same azimuthal location. Target trials were excluded from the analysis.

Sounds recorded in the virtual reality lab were presented to the subjects in the MRI scanner, creating the percept of space, using the MRI-compatible S14 model earphones of Sensimetrics Corporation (www.sens.com) with a linear frequency transfer up to 8 kHz. Before starting the experiment (with the ear buds in place), the subjects were instructed to adjust the overall sound intensity to a clearly audible and comfortable level. This resulted in an approximate sound intensity of 65 dB. Next, to ensure that subjects perceived sounds in the correct location, example sounds with a random order of the seven azimuthal locations were presented. We asked the subject to indicate where the sound originated from, and adjusted the ear buds until the subjects correctly localized these example sounds. Each training and testing run lasted ~9 and 13.5 minutes, respectively, resulting in ~81 minutes of functional data per subject.

Functional data were analyzed with BrainVoyager QX. Preprocessing consisted of slice scan-time correction (with sinc interpolation), 3-dimensional motion correction, and temporal high pass filtering (removing drifts of 4 cycles or less per run). Functional data were co-registered to the anatomical data, normalized in Talairach space^54^, and resampled (with sinc interpolation) at a resolution of 0.5 mm isotropic.

### Stimulus representation in the computational encoding model space

We compared the ability of four computational encoding models (a *frequency only* model, a *location only* model, an *independent frequency-location* model, and a *joint frequency-location* model) to represent the subcortical brain responses to the presented sounds. The representation of the training sounds in the *frequency only* model space was obtained as the output of the first (early) stage of a biologically inspired model of auditory processing^25^,^27^,^28^,^58^ (see top row of Fig. 1a; NSL Tools package, available at http://www.isr.umd.edu/Labs/NSL/Software.htm). This model mimics the spectral transformation of sounds passing through the cochlea to the midbrain, and includes a bank of 128 overlapping bandpass filters equally spaced along a logarithmic frequency axis (180–7040 Hz; range of 5.3 octaves). The spectrograms resulting as the output of this model were averaged over time. In order to avoid overfitting, we divided the tonotopic axis into 42 bins with equal bandwidth in octaves and averaged the model’s output (128 frequency bins) within these regions. The resulting number of 42 parameters to estimate was chosen based on the number of training sounds (56 sounds) and was kept stable across the four encoding models.

The stimulus representation in the *location only* model space was obtained by creating a [1 × *F*] vector for each sound (where *F* is the number of features or parameters to estimate), and setting it to [1/6 1/3 1 1 1/3 1/6] in those 6 bins representing the location in which the sound was presented (i.e. bin 1 to 6 for −90°, bin 7 to 12 for −60°, etc.; see bottom row of Fig. 1a). For the *independent frequency-location* model, the stimulus representation in the model’s space was created by concatenating the representation of the sound in the frequency space (reduced from 128 to 35 frequency bins) with a [1 × 7] location vector in which only the location representing the sounds’ location was set to [1] (see second row of Fig. 1a). Finally, the *joint frequency-location* model was obtained by setting the bins representing the location of the sound within a [1 × *F*] vector to the values obtained from reducing the sound’s frequency representation to 6 bins (see third row of Fig. 1a). As a result, each model consisted of a different number of frequency and location bins (42/0/35/6 frequency bins, and 0/42/7/7 location bins for the four models respectively), but had the same number of parameters to estimate. For each of the four models, the sounds’ representation in model space resulted into an [*S* × *F*] feature matrix **W**, where *S* is the number of training sounds.

### Estimation of the subcortical responses to the sounds

Independent training and testing runs (in which completely distinct sets of sounds were presented) were used to train and assess the models. We calculated the fMRI response **Y** ([*S* × *V*], where *V* = number of voxels) to the sounds in three steps. First, we computed noise regressors to denoise the data^59^ (http://kendrickkay.net/GLMdenoise/). These regressors were added to the second and third step, which were otherwise executed as described before^58^. That is, as a second step an optimized HRF per voxel but the same across sounds was computed using a deconvolution analysis. Third, the estimated HRF per voxel was used to estimate the amplitude of the response (i.e. beta weight) to each sound.

### Model parameter estimation and evaluation

Model estimation and evaluation was performed on the best 5000 voxels, provided that these voxels responded significantly to the sounds at the individual subject level (t > 2.9, *p* < 0.005 uncorrected) within an anatomical mask. The anatomical mask consisted of three rectangular boxes, positioned to include the IC, left MGB, and right MGB. We estimated each model’s parameters using customized Matlab code (www.mathworks.com). Based on the sounds feature representation **W** and the fMRI response matrix **Y**, the voxels’ feature tuning (matrix **R** [*F* × *V*], where *V* = number of voxels) was obtained as a solution to the linear problem:


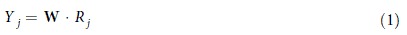


where each element *i* of the vector *R*_*j*_ describes the contribution of a specific frequency bin and/or azimuthal location *i* to the overall response of voxel *j*. The solution to Equation 1 was computed using ridge regression^28^. The regularization parameter λ was determined independently for each voxel by automatically inspecting the stability of the ridge trace^28^.

We assessed model performance as its accuracy in predicting responses to novel testing sounds^24^,^25^,^28^ (“sound identification analysis”). Namely, we used the estimated feature preference of each voxel *j* to predict the response *Ŷ*_*test,j*_ as:


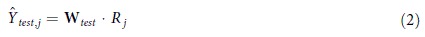


where **W**_***test***_ [*S*_*test*_ × *F*] is the representation of the testing sounds in the model space (*S*_*test*_ = 28). For each sound *i* we computed the correlation between the predicted response *Ŷ*_*test,i*_ [1 × *V*] and the measured fMRI responses to all testing sounds **Y**_***test***_. Rank *r*_*i*_ of the correlation between predicted and observed responses to sound *i* measures the models ability to correctly match predicted response *Ŷ*_*test,i*_ with measured response *Y*_*test,i*_. Thus, a rank of 1 indicates perfect prediction, while a rank of *S*_*test*_ represents the worst outcome. Prediction accuracy *P*_*i*_ of each sound was defined as 1 - the normalized rank:


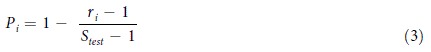


Values of *P*_*i*_ range between 0 and 1, with perfect prediction = 1 and chance = 0.5. The overall accuracy of the model was obtained as the mean prediction accuracy across all testing sounds. Statistical significance of the prediction accuracy at single subject level was assessed with permutation testing. That is, the empirical null-distribution was obtained by pseudo-randomly permuting the stimulus labels (*S* in matrix **Y**), while ensuring that the shuffled label did not originate from the same sound location as the original label, and repeating the analysis (200 permutations). The regularization parameter was constant across permutations and for each voxel was set to the value obtained from the unpermuted training analysis. Statistical significance of the prediction accuracy for each model was obtained at the group level by performing a one-tailed one sample t-test after Fisher transformation of the values.

### Topographic maps of frequency and spatial location

For the IC, optimization of anatomical across-subject alignment was performed on the posterolateral IC outline as defined on the T_1_/PD data. For the MGB, anatomical alignment was performed by running the automatic anatomy-to-anatomy alignment as implemented in BrainVoyager on the siT_1_-based manually segmented MGB. The automatic alignment was visually inspected and small manual adjustments were made where needed. The functional data was brought to this group-aligned space for the MGB and IC separately. For each dataset, a fixed effects General Linear Model (GLM) analysis created the final group maps (see Supplementary Fig. 2b for MGB group maps before and after siT_1_ alignment). Voxels outside the grey matter (i.e. vessels, CSF) were excluded in each individual.

Topographic maps were computed based on the *joint frequency-location* model, within those voxels that showed a significant response to the sounds at the group level (*p* < 0.01 uncorrected). For this purpose, group maps were transformed back to each individual space (applying the inverse transformation of the across subject alignment), such that voxel selection and topographic map computation was performed in Talairach space. For each voxel *j*, *R*_*j*_ was reorganized as a matrix of 6 frequencies by 7 locations and 2D smoothed (Gaussian filter; size = 3 points; SD = 0.5). The frequency and azimuth corresponding to the maximum of this matrix were used for creating maps of tonotopy and location respectively. Tonotopic maps were created by logarithmically mapping frequency values to a red-yellow-green-blue colour scale. Binary location maps were created by colour-coding preferred azimuth locations on the left and right in green and orange respectively. Contra-ipsilateral location maps were created by multiplying all azimuth values in the left subcortical regions by −1, such that all negative/positive values indicated a preference to contra- and ipsilateral sound locations respectively. Group maps were created by bringing individual maps to the siT_1_-aligned group space and subsequently averaging maps across subjects.

## Additional Information

**How to cite this article**: Moerel, M. *et al.* Processing of frequency and location in human subcortical auditory structures. *Sci. Rep.* **5**, 17048; doi: 10.1038/srep17048 (2015).

## Supplementary Material

Supplementary Information

## Figures and Tables

**Table 1:** Center of mass of low-high-low tonotopic pattern in MGB.

	Right MGB			Left MGB		
	X [SD]	Y [SD]	Z [SD]	X [SD]	Y [SD]	Z [SD]
Low DM	11.3 [1.4]	−25.7 [0.5]	−3.0 [0.6]	−13.7 [1.2]	−25.3 [0.8]	−2.3 [0.8]
High	13.2 [1.3]	−24.0 [0.6]	−3.7 [0.5]	−15.3 [1.4]	−24.3 [0.8]	−3.3 [0.8]
Low VL	14.3 [2.0]	−22.8 [1.0]	−4.0 [0.6]	−16.7 [1.6]	−23.5 [0.6]	−4.2 [1.3]

The Talairach center of mass (COM) of three regions (dorsomedial [DM] and ventrolateral [VL] low frequency regions, and high frequency region) within the MGB, averaged across individuals. COMs were defined separately for the left and right MGB.
