# Supplementary material for: Processing of frequency and location in human subcortical auditory structures
Source: Sci Rep. 2015 Nov 24;5:17048. doi: 10.1038/srep17048 (PMC4657019; doi:10.1038/srep17048)
Supplement: Supplementary Information [file srep17048-s1.pdf]

Processing of frequency and location in human subcortical auditory structures

Supplementary information

Michelle Moerel[1*], Federico De Martino[1-3], Kâmil Uğurbil[1], Essa Yacoub[1], Elia Formisano[2-3]

[1]Center for Magnetic Resonance Research, Department of Radiology, University of Minnesota, Minneapolis, USA

[2]Department of Cognitive Neuroscience, Faculty of Psychology and Neuroscience, Maastricht University, Maastricht, the Netherlands

[3]Maastricht Brain Imaging Center (MBIC), Maastricht, the Netherlands

***Corresponding author:**

Michelle Moerel

Center for Magnetic Resonance Research, Department of Radiology,

University of Minnesota

Minneapolis 55455 MN, USA

mlmoerel@umn.edu

Phone: +1 612 626 2001, Fax +1 612 626 2004

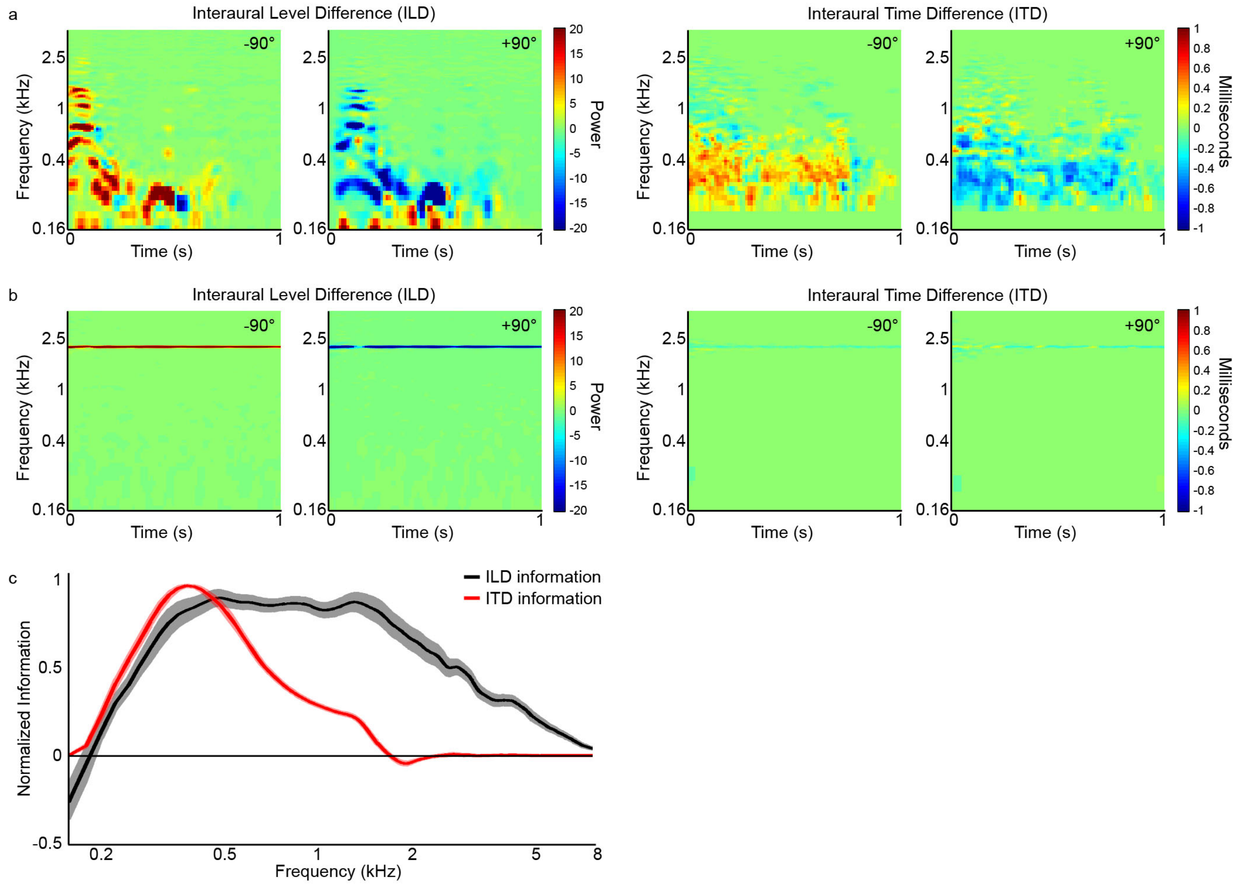

Supplementary Figure 1. Characterization of the stimuli.

(a-b) Interaural level difference (ILD) and interaural time difference (ITD) for two recorded sounds presented on the left (-90°) and the right (+90°) of a representative subject. A speech utterance (containing mostly low frequencies) and a bell sound (with exclusively high frequency content) are shown in a and b, respectively. (c) ILD and ITD information per frequency bin, averaged across 84 sounds recorded separately for 6 subjects (mean ± s.e.m. across subjects). ILD (or ITD) information was computed as the normalized difference between the ILD (or ITD) at +90° and the ILD (or ITD) at -90°. Positive values indicate that the ILD or ITD cue is informative. ITD cues provided spatial information only in lower frequency ranges (between 0.2 and 1.5 kHz), while ILD cues were informative for all frequencies above 0.3 kHz (alpha level = 0.05; N = 6; one-tailed one sample t-tests corrected for multiple comparisons). The ILD information in low frequencies is likely due to the presence of reverberations in the virtual reality lab.

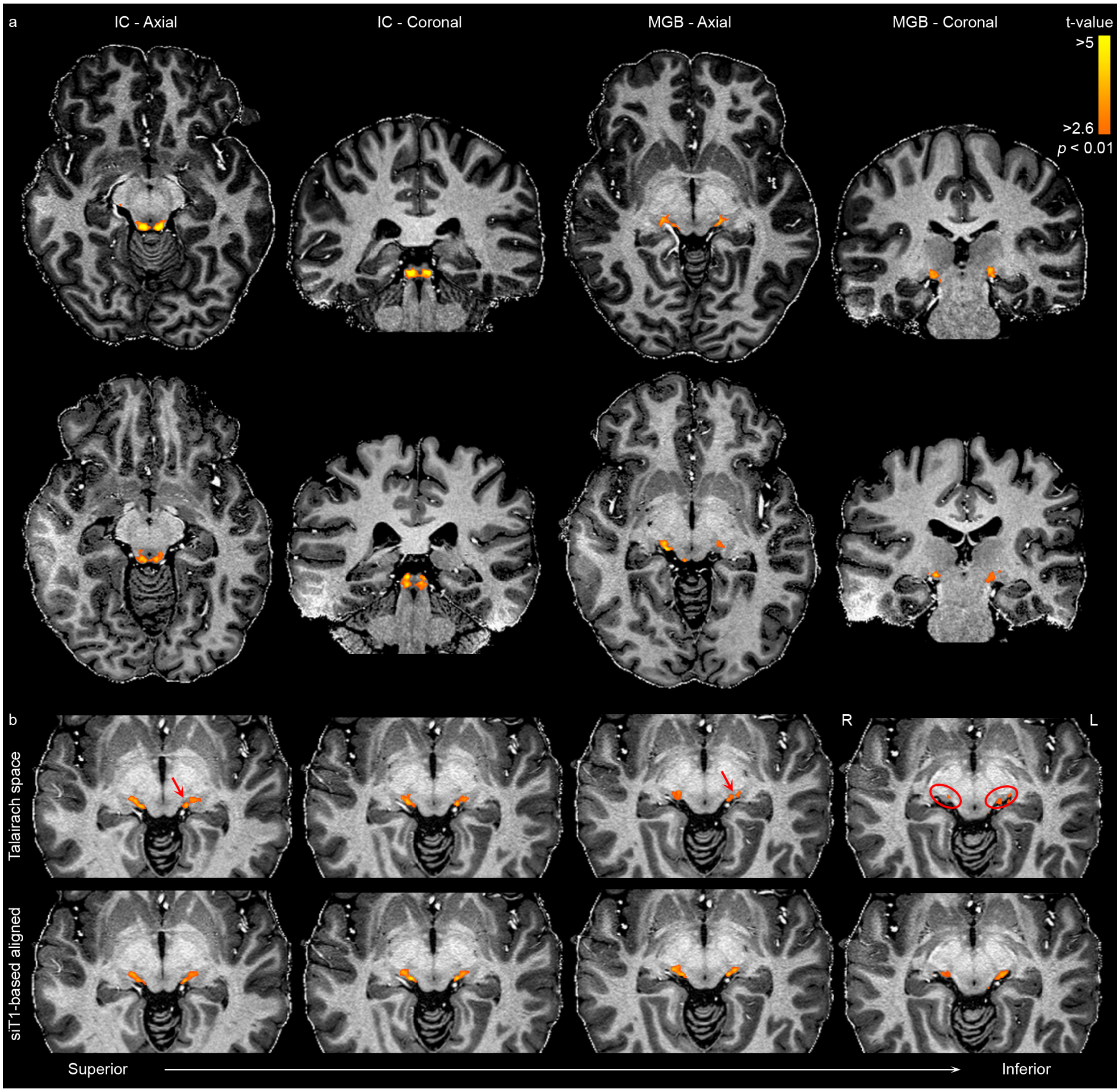

Supplementary Figure 2. Functional responses in the IC and MGB.

(a) Overall response in selected axial and coronal slices in subject 1 (top) and subject 6 (bottom). Maps are cleaned with a grey matter mask and thresholded at $p < 0.01$ (uncorrected). They are spatially smoothed with a Gaussian kernel (FWHM = 3 voxels) for display purposes only. (b) Alignment of the MGB based on the siT_1 data. Axial slices, ranging from superior (left) to inferior (right), with group responses computed after transforming each individual dataset to Talairach space (top) and after siT_1-based MGB alignment (bottom) are shown. Differences across alignment methods can be observed in the continuity of the left MGB (red arrows) and the response in inferior MGB locations (red circles).

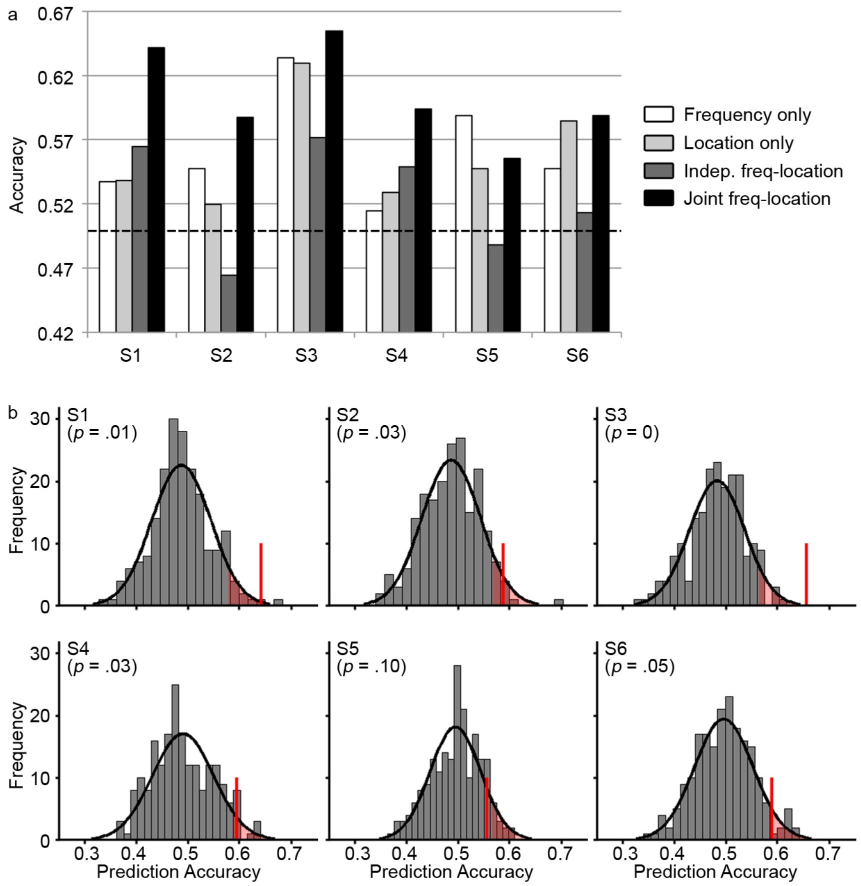

Supplementary Figure 3. Model performance in individual subjects.

(a) Bars indicate the prediction accuracy for the four models in each individual. Chance level is 0.5 and is indicated by the dashed black line. (b) The histograms reflect the prediction accuracies obtained from 200 permutations of the labels (i.e. sounds S in matrix of fMRI responses $\mathbf{Y}$) for each subject separately. The normal curve is defined by the mean and SD of the underlying distribution. The red shading reflects the 95% confidence interval. The red marker reflects the prediction accuracy from unpermuted labels based on the *joint frequency-location* model. The p values reflect a significantly above chance prediction accuracy (alpha level = 0.05) in five out of six subjects.

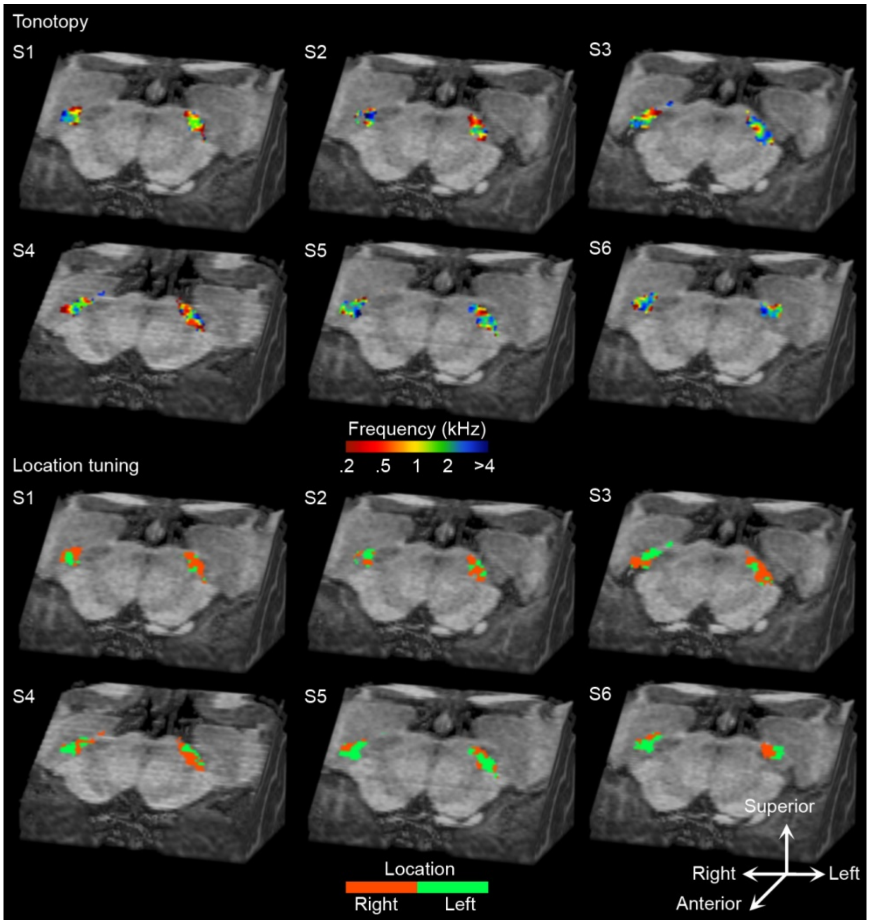

Supplementary Figure 4. Individual frequency and location preference in the MGB.

Maps of frequency preference (tonotopic maps; top) and azimuth preference (tuning to spatial location; bottom) in the MGB for all subjects. A cube of brainstem based on a high-resolution anatomical image of the subject is shown from the front-left, and a cut through it is made at a 45° angle such that the MGB is visible. The maps are superimposed on this image.

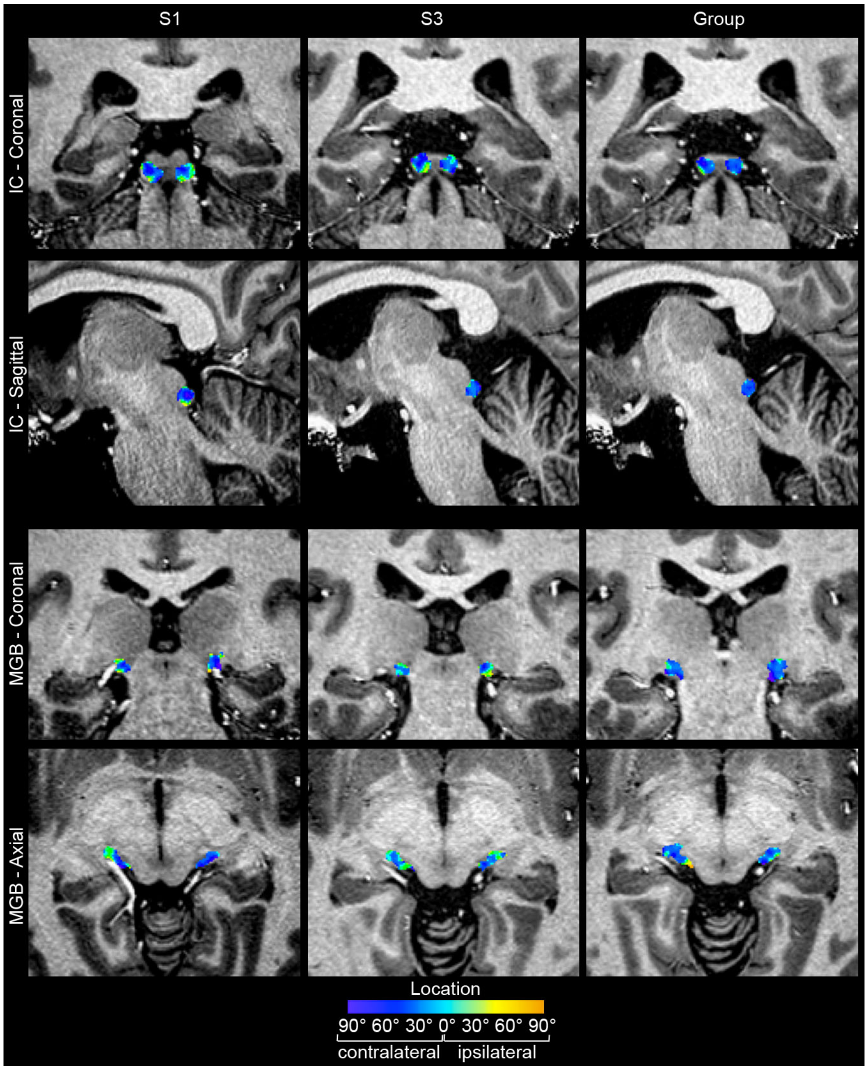

Supplementary Figure 5. Maps of preferred location in the IC and MGB.

Maps of preferred spatial location in the IC (top) and the MGB (bottom) are shown for two representative individuals (S1 and S3, first and second column respectively) and in the group (third column). Coronal and sagittal views are shown for the IC (first and second row), and coronal and axial slices are displayed for the MGB (third and fourth row). Values in the left IC and MGB are multiplied by -1, such that the maps reflect preference to the ipsilateral or contralateral hemifield in orange/yellow and purple/blue respectively. Maps show an overall bias to the contralateral hemifield (reflected by the predominance of purple/blue colours) with ipsilateral regions tending to occur on the periphery of the nuclei. No topographic gradient of preferred location was observed.

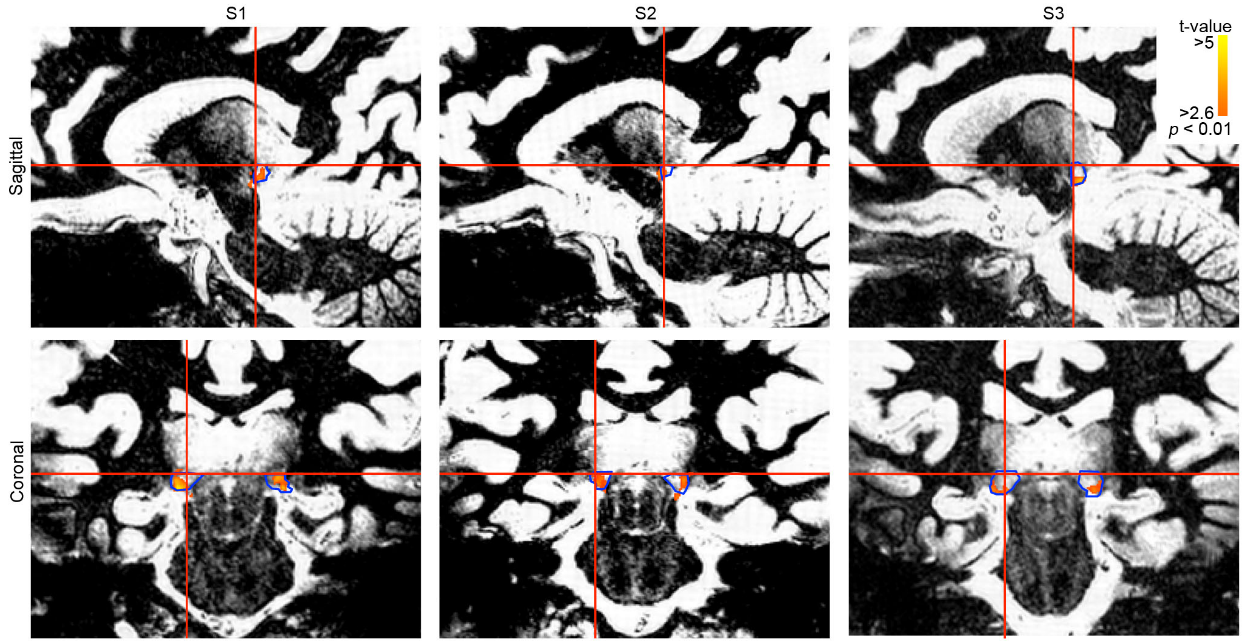

Supplementary Figure 6. Correspondence between functional responses and siT_1-defined MGB.

Orange colours and blue outlines show the overall MGB response to natural sounds ($p < 0.01$ uncorrected) and the siT_1-based delineation of the MGB in three individual subjects. The red lines indicate the same Talairach coordinate across subjects and viewpoints ([x y z] = [13 -24 0]; right MGB). The functional responses occupied the inferior (visible in sagittal slices; top row) and lateral (visible in coronal slices; bottom row) part of the siT_1-defined MGB. This relationship between functional and anatomical results was stable and held across variations present at the level of individual subjects (compare e.g. coronal slices of S2 and S3; the siT_1-based MGB extends inferior in S3 compared to S2, and the functional responses follow this offset). Note that these slices were chosen to display the correspondence between function and anatomy, and are not representative for the extent of IC activation (see Supplementary Fig. 2 for slices through the middle of the IC).
